# The Interaction of FABP with Kapα

**DOI:** 10.1371/journal.pone.0132138

**Published:** 2015-08-18

**Authors:** Ortal Amber-Vitos, Nataly Kucherenko, Esther Nachliel, Menachem Gutman, Yossi Tsfadia

**Affiliations:** Department of Biochemistry and Molecular Biology, Tel Aviv University, Ramat Aviv, Tel Aviv, Israel; Weizmann Institute of Science, ISRAEL

## Abstract

Gene-activating lipophilic compounds are carried into the nucleus when loaded on fatty-acid-binding proteins (FABP). Some of these proteins are recognized by the α-Karyopherin (Kapα) through its nuclear localization signal (NLS) consisting of three positive residues that are not in a continuous sequence. The Importin system can distinguish between FABP loaded with activating and non-activating compounds. In the present study, we introduced molecular dynamics as a tool for clarifying the mechanism by which FABP4, loaded with activating ligand (linoleate) is recognized by Kapα. In the first phase, we simulated the complex between Kapα^ΔIBB^ (termed “Armadillo”) that was crystallized with two NLS hepta-peptides. The trajectory revealed that the crystal-structure orientation of the peptides is rapidly lost and new interactions dominate. Though, the NLS sequence of FABP4 is cryptic, since the functional residues are not in direct sequence, implicating more than one possible conformation. Therefore, four possible docked conformations were generated, in which the NLS of FABP4 is interacting with either the major or the minor sites of Kapα, and the N → C vectors are parallel or anti-parallel. Out of these four basic starting positions, only the FABP4-minor site complex exhibited a large number of contact points. In this complex, the FABP interacts with the minor and the major sites, suppressing the self-inhibitory interaction of the Kapα, rendering it free to react with Kapβ. Finally, we propose that the transportable conformation generated an extended hydrophobic domain which expanded out of the boundary of the FABP4, allowing the loaded linoleate to partially migrate out of the FABP into a joint complex in which the Kapα contributes part of a combined binding pocket.

## Introduction

Fatty acids and their related compounds are often associated with gene regulation [1, 2]. Transport of these compounds across the nuclear membrane is mediated by fatty acid binding proteins, FABPs, which are identified by the Karyopherin system (Importin system) [3, 4]. The FABP molecules share a common structure: ten β-strands, forming a β-clam configuration that encloses an inner space in which the fatty acid ligand is embedded. The hydrophobic β-clam domain is covered by two α helices, connected by a short loop, forming a “lid”. In order to transport these ligands across the nuclear membrane, the FABPs generate a homodimeric structure that exposes the lid and its NLS residues to the bulk, thus allowing them to interact with Kapα [5].

**Fig 1:**
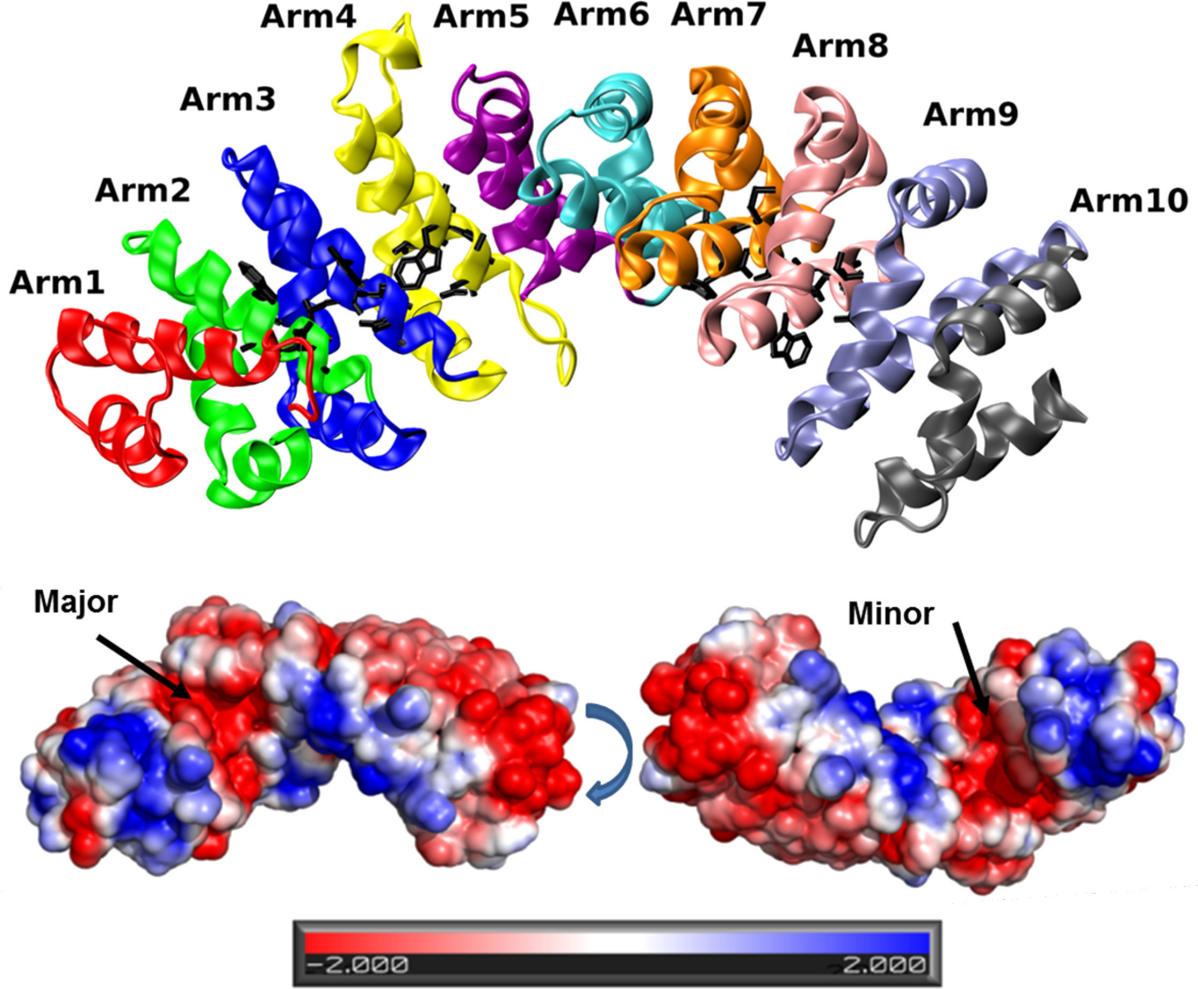
Upper Frame: The structure of the Kapα Armadillo colored by its Arms. The WxxxN moieties of the major and minor sites, which are located on Arms 2, 3, 4 and 7, 8, respectively, are shown as black sticks. The lower Frames depict the electrostatic potential of the Armadillo, demonstrating the negative patches of the major and the minor sites, using the Pymol APBS tool [38–40]. The two faces of the protein are presented by rotation 180^0^ of the protein, along its long axis.

Usually, the residues forming the NLS recognition site are composed of three positive amino acids. In the "classical signal" case, the residues are KK/RXK/R, typically appear as a continuous section. However, some proteins are marked by a cryptic signal when a section of 8–10 residues [6], is inserted in the middle of the NLS. Some members of the FABP family carry an NLS sequence, which is also cryptic in nature [7–9]. In the case of FABP4, it consists of one residue K21 located near the C terminal side of helix 1, while the other two moieties (R30 and K31) are near the N terminal section of helix 2, with a short unstructured loop between them (as well as K24, R33, K34 in FABP5)[10]. The proteins that recognize the NLS and offer a transport mechanism into the nucleus are members of the Karyopherin system (Kapα and Kapβ) [3, 11–13]. Kapα is a long protein made of an N terminal section termed IBB (Importin β Binding domain) and a set of 10 repeating structures, each consisting of 3 α-helices (H1, H2, H3),[4], that are assembled in a banana-shaped structure termed the "Armadillo". On the Armadillo there are two sites that can identify an NLS sequence termed the major and the minor sites, each having a specific sequence WxxxN that is involved in the binding of the NLS. The major site is located on Arms 2, 3 and 4 interacts with the three moieties of the NLS (see Fig 1). The minor site is located on Arms 7 and 8, and interacts with only two residues of the NLS, as demonstrated in Fig 1. Generally, the major site has a higher affinity to form complexes [3, 4], yet there are some proteins that prefer the minor site [11]. Kapα proteins bind their cargo via an NLS signal and react through their IBB domain with Kapβ. The whole complex is carried into the nucleus through the nucleoporins.

The IBB has another function; due to an NLS sequence located on the IBB, it can cover the major site of the Armadillo. In this folded conformation, a "self-inhibited" state, Kapα is not recognized by Kapβ and cannot be transported into the nucleus. This feature is instrumental in identifying a putative functional FABP4-Kapα complex; its structure should prevent the formation of the self-inhibited shape.

The FABP4-Kapα import system is very selective. The complex is formed only with FABP4 loaded with a certain substrate molecule. Thus, FABP4 will be carried into the nucleus when loaded with linoleate but not if the ligand is palmitate. This selectivity is attributed to the shape of the FABP4 dimer; in the case of the non-transportable compounds, the contact between two FABP4 molecules in the dimer is at the lid zone, a conformation that masks the NLS domain of the FABP. FABP molecules that are loaded with transportable ligand form the dimer by joining the other pole of the molecule, leaving the lid fully exposed and available for reacting with the Armadillo [5]. Once the loaded FABP4 molecule is located in the nucleus, it releases its cargo that reacts with the PPAR (Peroxisome Proliferator Activator receptor) RXR (Retinoic X Receptor) system, which is a cardinal gene expression machinery that controls the carbohydrate-lipid metabolic balance [14–18]. Considering that the affinity of FABP to a fatty acid is in the sub-micromolar range [19–21], the mechanism of the fatty acid release inside the nucleus is also an intriguing problem.

The complexes of the NLS peptides with Kapα were investigated through crystallization of the Kapα^ΔIBB^ (only the Armadillo) with short peptides, bearing the NLS sequence. In the complex of Armadillo with the SV40 large T antigen (PKKKRKV), two peptides are bound at the major site (the positive residues of the peptide are termed P1 to P5 [22]) and at the minor site (termed P1’ to P4’, starting from the second lysine [22]) (1EJL.pdb). The peptide molecules at either site have no secondary structure and their interactions with the Armadillo are both through their backbone atoms and side-chain moieties.

The identification of the NLS residues on FABP4 [8–10, 23, 24] was confirmed by the replacement of all the three moieties by alanine. This gross replacement experiment cannot exclude the possibility that the FABP4 may interact with only two residues with the minor site of Kapα, as in the case of the human phospholipid scramblase [11]. Consequently, we have no assurance whether the FABP4 binds to the major or the minor sites of Kapα, nor the interaction direction (the N → C vectors) parallel or anti parallel.

The interaction between the FABP4 with Kapα takes place in dilute electrolyte solution, conditions that grossly differ from the crystallization environment, suggesting that the interaction between the two proteins in solution will involve conformations that are not identical to the crystalline state of the proteins. In the present study we investigate, by all atom molecular dynamics, how the NLS domain of the FABP4, loaded with linoleate, interacts with the major or minor NLS binding domain of Kapα. For that purpose we first simulated the "simple" system of the SV40 peptides bound to Armadillo (crystal structure – 1EJL.pdb [25]), examining the rigidity of the peptide's structure and the contribution of each of the residues to the stability of the complex.

The results indicate that even when the simulations were initiated from a very stable crystalline structure, within a short time the peptides in both sites deviated from their original conformations. Moreover, even that the overall intensity of the interactions (Lennard-Jones and electrostatic) between the two reactants was rather stable with time, the relative contribution of each of the 7 residues of the peptides alternated with time. The recognition that even the minimal system has more than one stable form suggests that the FABP-Armadillo complex may also be stabilized by more than a single set of contacts. The strategy selected to study the FABP4-Armadillo complex was based on the biological evidence that established the role of the NLS moieties of FABP4 as the recognition elements that interact with the Armadillo [8–10, 23, 24], and looked also for interactions between residues that are not *bona fide* members of the NLS or the WxxxN sequences. Based on these observations, we reconstructed a possible mode of interaction between the FABP4 with the Armadillo, testing both major and minor sites in parallel and anti-parallel directions. The results presented below imply that the most likely location for the FABP4 to interact with Kapα is at the minor site of the Armadillo, where the N → C vectors are parallel. In this conformation, we observed many non-NLS moieties that maintain stable contacts with the residues on both major and minor sites, preventing the IBB from folding over, and rendering the complex unrecognizable by Kapβ.

## Methods

The simulations of the Armadillo complex with NLS of the SV40 large T antigen (PKKKRKV) were based on the crystal structure from the Protein Data Bank (pdb code 1EJL [25]).

For the complex of the Armadillo with the FABP4 loaded with linoleate, we replaced the short NLS peptide, using the crystal structure of the FABP4 loaded with linoleate (2Q9S.pdb [5]). We set the positive moieties (K21, R30, K31) near Arms 2, 3 and 4 (for interaction with the major site) and near Arms 7 and 8 (for interaction with the minor site) of the Armadillo. Care was taken to avoid clashes between the two proteins. The procedure was repeated twice, where the N → C vectors of the two proteins were in parallel and anti-parallel orientations. Following this initial setting, the systems were relaxed and simulated as described below.

### Molecular Dynamics and Simulation Programs

Standard MD simulations were carried out using of the GROMACS 4.0.7 package [26] with the GROMOS96 force field, with a 53a6 parameter set [27].

The parameters of the linoleate molecules (bond length, angles and dihedrals, improper and partial charges) were taken from the GROMACS standard building blocks for glutamate. The parameters of the double bond are GROMACS parameters for retinol as present in RTOL. The net charge of the ligands was -1 electron charge.

Prior to the MD step of each simulation, a dodecahedron box was built, with dimensions extended at least 12 Å from the nearest molecule, and was filled with water using the SPC216 model. Charge neutralization, while maintaining an ionic strength of approximately 100 mM, was obtained by adding Na^+^ and Cl^-^ ions. During the MD simulations, the LINCS algorithm [28] was used to constrain the lengths of all bonds; the water molecules were restrained with the use of the SETTLE algorithm [29]. All simulations were carried out under NPT conditions of constant number of moles, pressure and temperature, using the v-rescale coupling algorithm for keeping the temperature constant and Berendsen’s coupling algorithm for keeping the pressure constant (P = 1 bar; τp = 0.5 ps; T = 300 K; τT = 0.1 ps) [30]. A 12 Å cutoff was used for the Van der Waals (VdW) interactions. The long-range electrostatic interactions were treated by the Particle mesh Ewald (PME) [31]. Each system was relaxed by energy minimization using the steepest descent and the Conjugated Gradient methods (as embedded in the GROMACS package). The resulting structures then underwent 40 ps of simulation with the protein's position restrained, allowing the solvent (water) molecules to reach equilibrium. The system was simulated further for 0.2 ns of unconstrained equilibration simulation. After the equilibration it was possible to start generating the MD simulations with a 2 fs time-step, by which Newton's equations of motion were solved. Every simulation had a different initial velocity for each atom that was randomly generated from the Maxwell-Boltzmann distribution at 300 K. Snapshots were saved at 2 ps intervals. Root Mean Square Deviation (RMSD) and Root Mean Squared Fluctuation (RMSF) calculations were made for the backbone atoms.

### Docking of FABP4 on the Armadillo

The Armadillo (1EJL.pdb) and FABP (2Q9S.pdb) were docked using the PatchDock server [32–34]. The structures with the highest score were used for the simulations. The strategy for docking was set to fulfill the following requirements: at least two residues (K21, K30, R31) of the FABP4 must be docked with the Armadillo's WxxxN stretches (142–146, 184–188, 231–235 for the major site), or 357–361, 399–403 (for the minor site), while these residues should be within 5 Ǻ from the tryptophan moieties of these stretches. At this range the supposedly interacting residues are close enough to establish electrostatic interactions, yet are separated by at least one water molecule, which ensures structural flexibility as the two proteins interact.

### Structural and Energetic Analysis

The first step of this analysis was the generation of a full dataset of minimum distances between each pair of residues of the two structures, by the command, “g_mindist” of the GROMACS 4.0 package. The set was filtered to remove all pairs whose geometric mean value, calculated over a given time interval (few tens of nanoseconds) exceeded a cutoff of 4 Å.

Cluster analysis was performed for all the simulations by the command, “g_cluster” of the GROMACS 4.0 package. The analysis was performed using the Gromos algorithm with a cutoff value set to generate a sufficient number of clusters while maintaining statistical significance [35].

For calculating the interacting energies of the proteins or residues-proteins, "g_energy" command of the GROMACS package was applied. The Constrain Network Analysis (CNA) was used for the calculation of the Flexibility index parameters. The data was executed on the CNA web-server [36, 37].The electrostatic potential mapping of the protein's surface was calculated by the PYMOL–APBS plugin [38–40].

Hot Spots analysis was carried out using the KFC Server [41].

Principle Component Analysis (PCA) [42] was carried out by using the "g_covar" command of the GROMACS package. For extracting the Eigen vectors and analysis, we applied the "g_anaeig" command of the GROMACS package.

The analysis of Hydrogen bonds was performed using the web server of Tina and coworkers [43].

## Results

### The solution structure of the Armadillo complex with the SV40 large T antigen PKKKRKV

**Fig 2:**
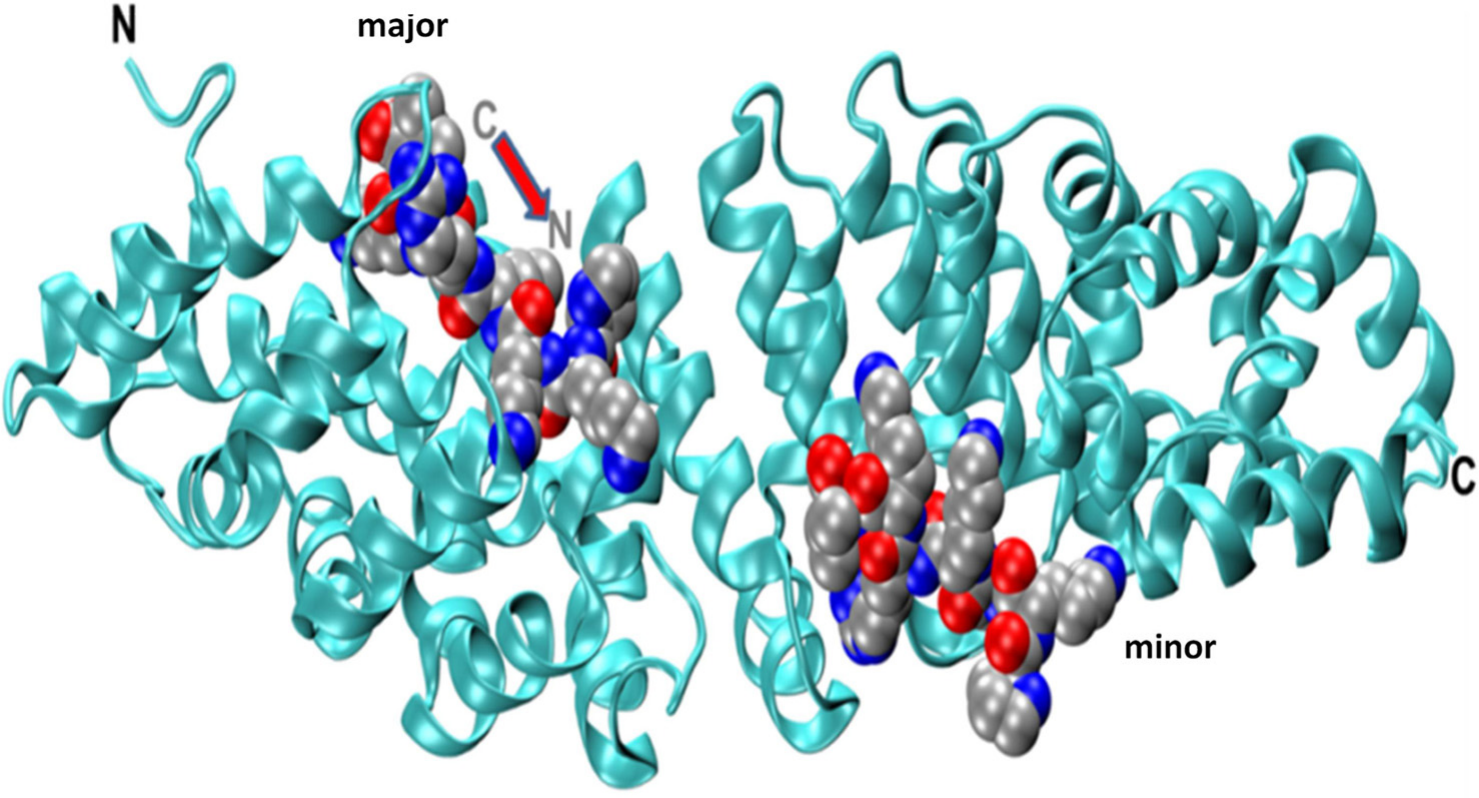
The crystal structure of the complex between Armadillo and two SV40 large T antigen PKKKRKV peptides (1EJL.pdb). The N-terminal IBB domain of the Kapα, consisting of a 69 residues, was removed prior to crystallization. The two peptides (shown in VDW representation, are located at the major (left) and minor (right) sites. The C→N axis of the two peptides, as marked for the peptide at the major site, are antiparallel to that of the Armadillo.

Fig 2 presents the crystal structure of the 10 Arms long Armadillo (the N terminal IBB domain was removed prior to crystallization), which is oriented with its N-terminus on the left and the two NLS peptides (PKKKRKV). The peptide on the left interacts with the major site of the Armadillo and the peptide on the right is bound to the minor site. The N → C terminal vector of the peptides is anti-parallel to that of the Armadillo. This complex was simulated three times for ~ 50 ns. For comparison, we also simulated twice the Armadillo in the absence of the NLS peptides. Examination of the RMSD and RMSF traces indicates that the binding of the peptides had a minor effect on the stability of the Armadillo, mostly decreasing the RMSF of the residues with which the peptides interact. However, the conformation of the hepta-peptide is affected by its interactions with the Armadillo during the simulation time. Under the stress applied by the crystallization setup, only one conformation is prevailing, but once the complex is placed in less restrictive medium, the hepta-peptide is fluctuating between seemingly equipotential conformations.

**Fig 3:**
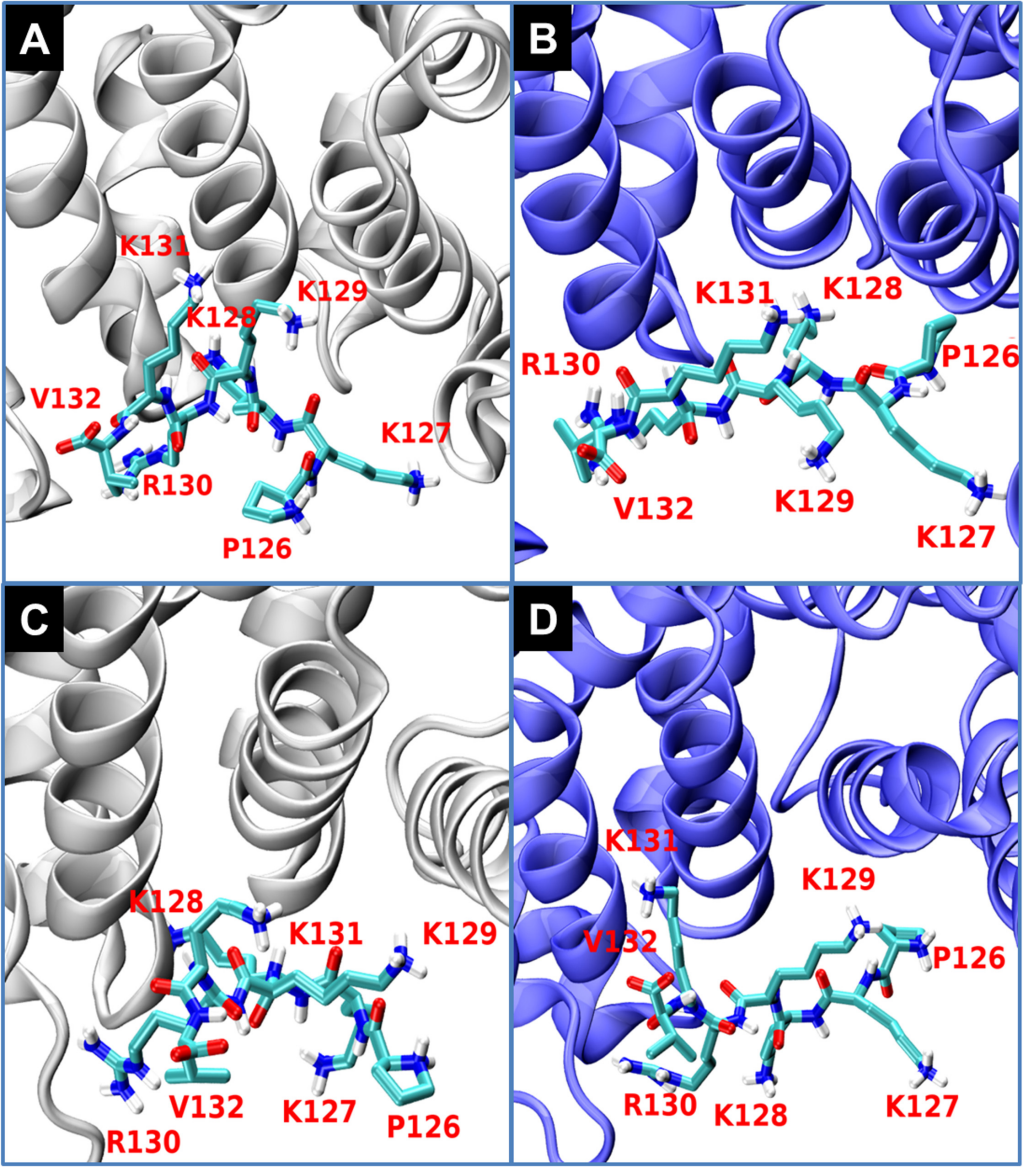
The NLS hepta-peptides conformation evolving over time. The initial (t = 1 ns) (Frames A, C–colored in grey) and final (t = 50 ns) (Frames B, D–colored in purple) structures of the NLS hepta-peptide located at the major (Frames A and B) and the minor (Frames C and D) sites of the Armadillo are shown. The NLS hepta-peptides residues are drawn as CPK coloring bonds and the Arms of the Armadillo are shown in a new-cartoon drawing style.

Fig 3 depicts the initial (Frames A and C) and final (Frames B and D) conformations of the hepta-peptide in the major (Frames A and B) and the minor sites (Frames C and D). These presentations clearly reveal that the orientation of each of the peptides prevailing in the crystal is changed and new interactions between the side chains and the Armadillo are formed. At both sites, the residues P126 and K127 are rotated as the complex assumes its solution structure. Similarly, the side chains of K128 and V132 also change their location. The structural fluctuations are not limited just to the side chains: as the peptides assume their new conformations, the backbone atoms of each of the peptides are shifted during the simulations. It should be notified that these conformational changes are not related with the B factor assigned to the two peptides in the crystal structure.

To quantify the conformational space sampled by each peptide, their trajectories were subjected to cluster analysis using a cutoff value of 0.5 Å. Of the two binding sites, the major site imposed more restrictions on the conformations of the peptide than the minor one. Accordingly, for the same cutoff value, 77 clusters characterizing the ligand at the minor site were found, while at the major site, the structure was much stable and only 27 clusters were identified.

**Fig 4:**
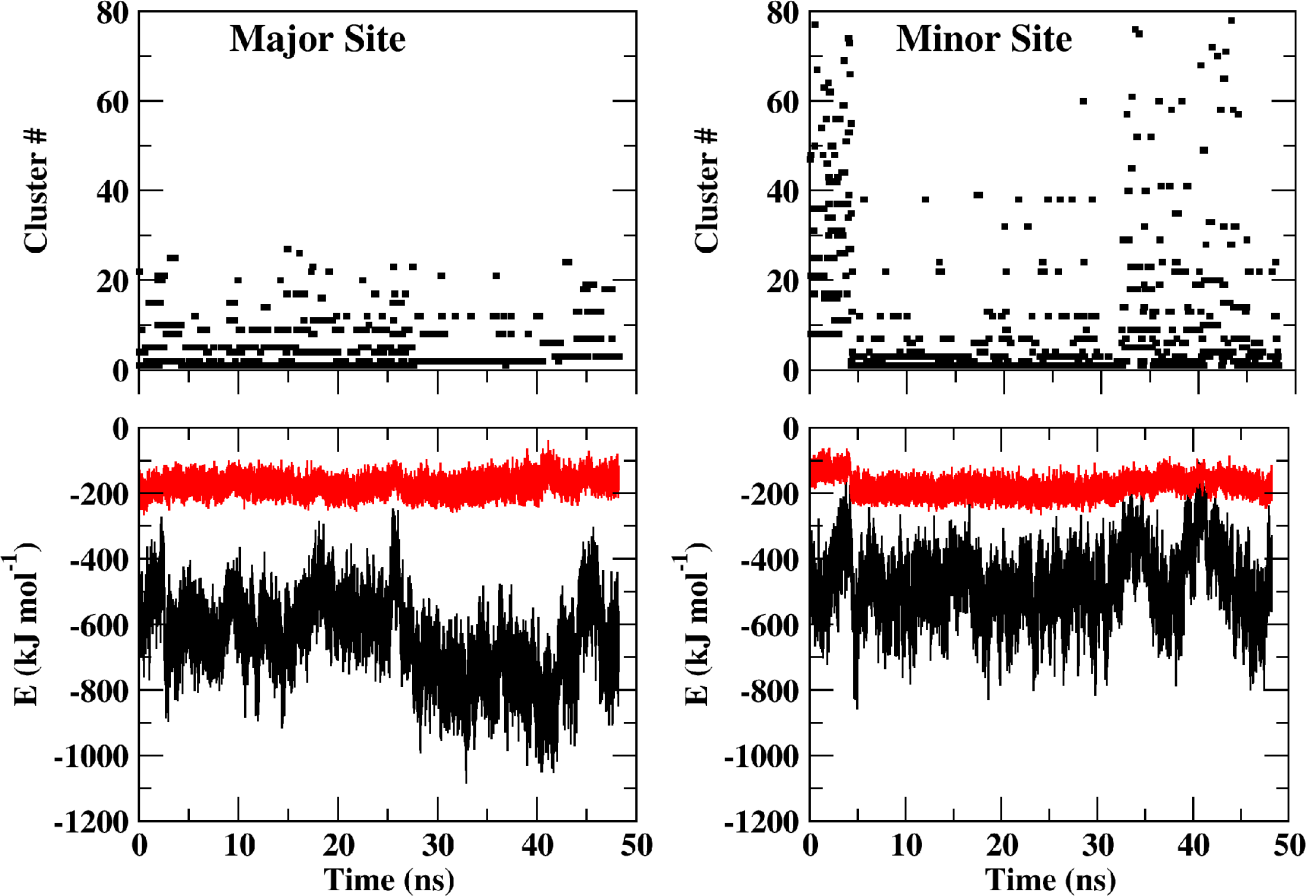
Temporal distribution of clusters representing the structure of the peptide at the major (left) and the minor (right) sites. The upper frames denote the temporal distribution of the clusters where high values correspond with low probability of the cluster. The lower frames present the electrostatic (black) and Lennard-Jones (red) interaction energies between the peptide and the Armadillo. The interaction energies and the cluster number are presented on the same time scale. To reduce the noise, the energies were smoothed by a running-average over a window of 10 ps.

In the upper frames of Fig 4, the temporal prevalence of the various clusters at the major and the minor sites (left and right, respectively) are presented. Each point corresponds with a representative structure of a cluster, where high numbers imply rare conformations. The lower frames present, over the same time axis, the intensity of the Lennard–Jones and electrostatic interactions between the peptides and the Armadillo. Comparison of the cluster distribution and the energy traces reveals a correlation between the two parameters. Thus, for the peptide bound to the minor site, we observe that during the first ~ 4 ns of the trajectory, the peptide sampled many conformations and none of the structures was dominating. We refer to this phase as an 'agitated-state', where both high probability clusters and rare conformations appear at comparable frequencies. At ~ 4 ns, there is a change in the regime, and a relaxed phase is noticed, where the high-probability conformations are dominant and low probability structures are rare. The agitated-relaxed states seem to be in a dynamic equilibrium as the transition had recurred more than once during the simulations (at t ~ 32 ns and ~ 42 ns). The interaction of the peptides with the major site is more intensive in terms of energy (notice the difference between the major and minor sites in the lower frames) and the total number of clusters (using the same cutoff value) was significantly smaller (27 *vs*. 77). The same pattern of alternating between relaxed and agitated phases is also common for the peptide attached to the major site; the peptide assumes its relaxed state between ~27 and ~41 ns of the simulation.

**Fig 5:**
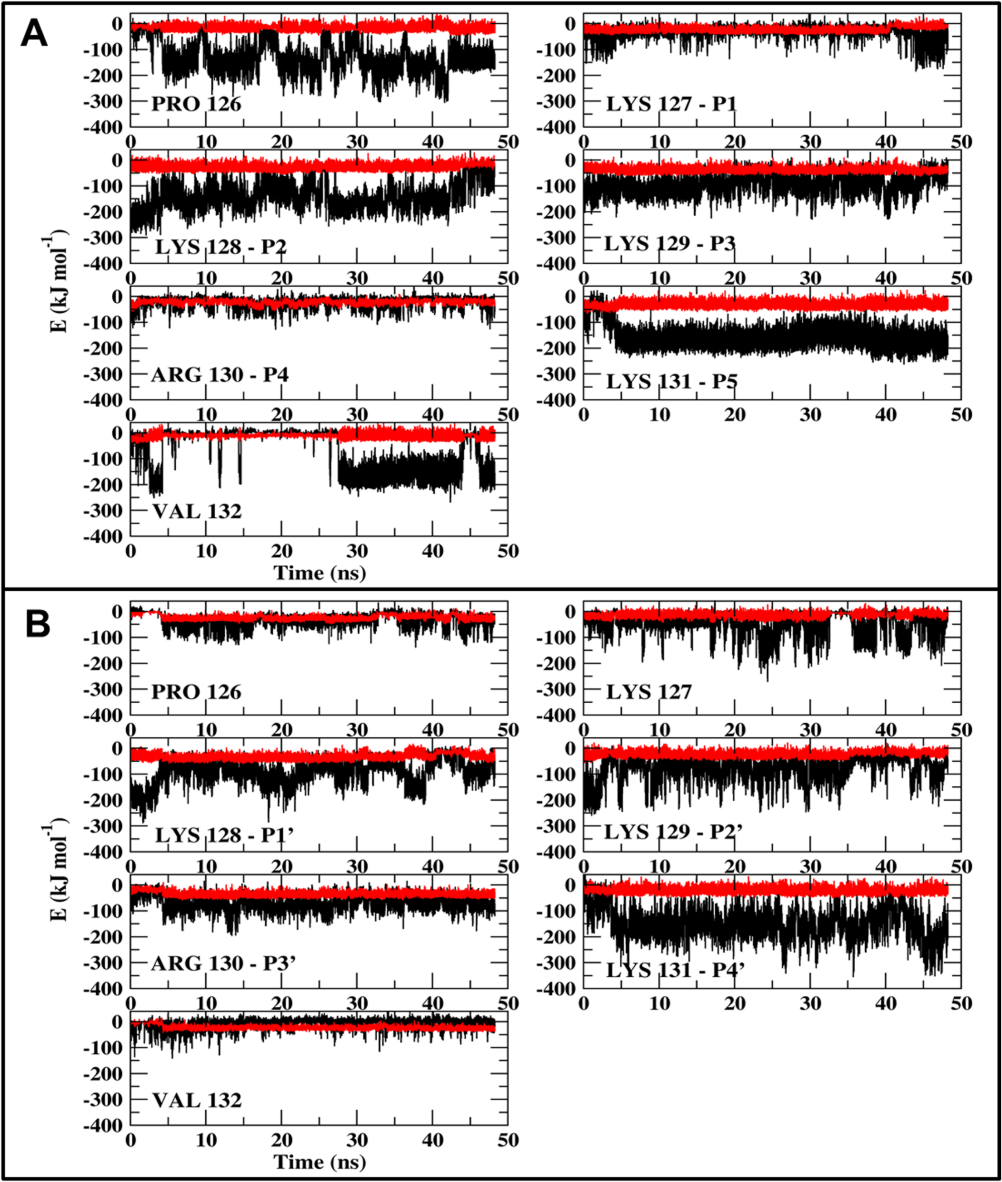
The interaction energies between each residue of the NLS peptide and the Armadillo. (A) depicts the interactions with the major site. (B) depicts the interactions with the minor site. The residues' identity is marked in the figure.

The variation in the intensity of the peptide-Armadillo interactions and the rapid shift between the conformations suggest that the charged moieties of the peptide fluctuate between more than one location. This feature was investigated by calculating the interacting energies of each one of the residues of the peptide with the Armadillo. The electrostatic interaction between the various residues of the peptide and the Armadillo can be as high as ~ -150 ± 20 kJ/mol per-residue (Fig 5). Yet, the interactions are not constant in time; at the major site (Fig 5, frame A) all of the residues contribute to the binding, but at each time point the identity of the stabilizing moiety is different. Generally, the interactions at p1, p3 and p4 are rather weak, while p2 and p5 are well binding, yet the intensity is subjected to large fluctuations. Surprisingly, both proline and valine, which are not a part of the conserved NLS sequence, contributed substantially to the stability. Two of the charged residues, K127 and R130, hardly contribute to the stability and the intensity of the electrostatic component of their contribution is not much larger than that of the Lennard-Jones interaction. The other charged moieties participate in intensive interactions, but at certain time frames the positive moieties exchange the intensity of the electrostatic interactions with the Armadillo by exposure to the solvent without reducing the total interactions with the Armadillo.

At the minor site (Fig 5, frame B) we also observe temporal fluctuations in the intensity of the electrostatic interactions. The interactions of the Armadillo with K128, p1’, p2’ and p4’ are intense and fluctuate with time, alternating from ~ -10 kJ/mol (comparable with the level of Lennard-Jones attraction) to ~ -200 kJ/mol. The interaction with p3' is stable but less intensive.

**Fig 6:**
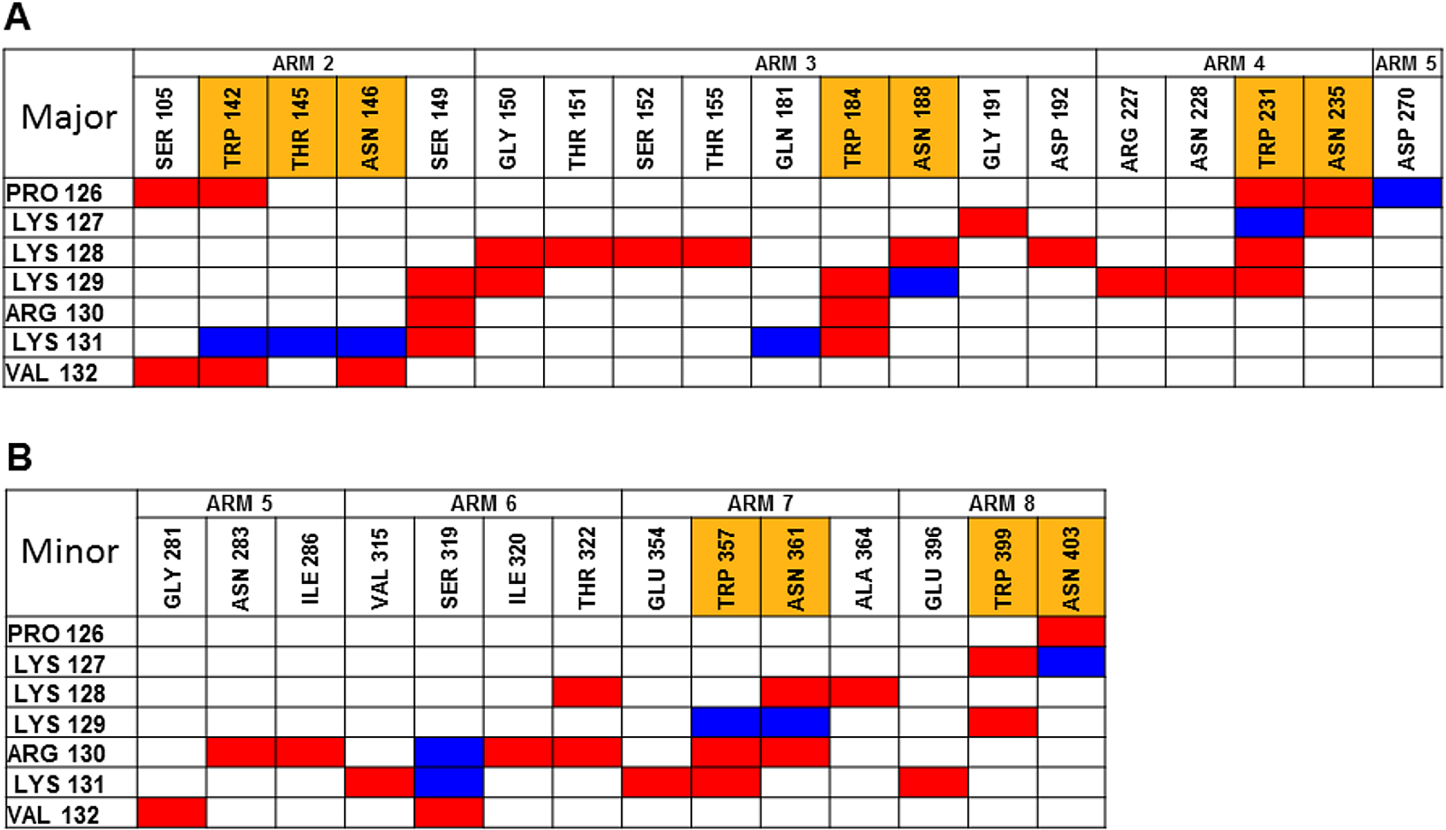
Identification of the stable residue pairs holding the NLS peptide at the major site. The last 30ns of the trajectory were analyzed in a search for pairs that maintained close contact with each other. The residues of the Armadillo and the NLS are grouped according to the Arm number. Residues located on the WxxxN repeat are marked in orange. Residues less than 4 Å or 2.8 Å apart are colored in red and blue, respectively. (A) depicts the interactions with the major site. (B) depicts the interactions with the minor site.

**Fig 7:**
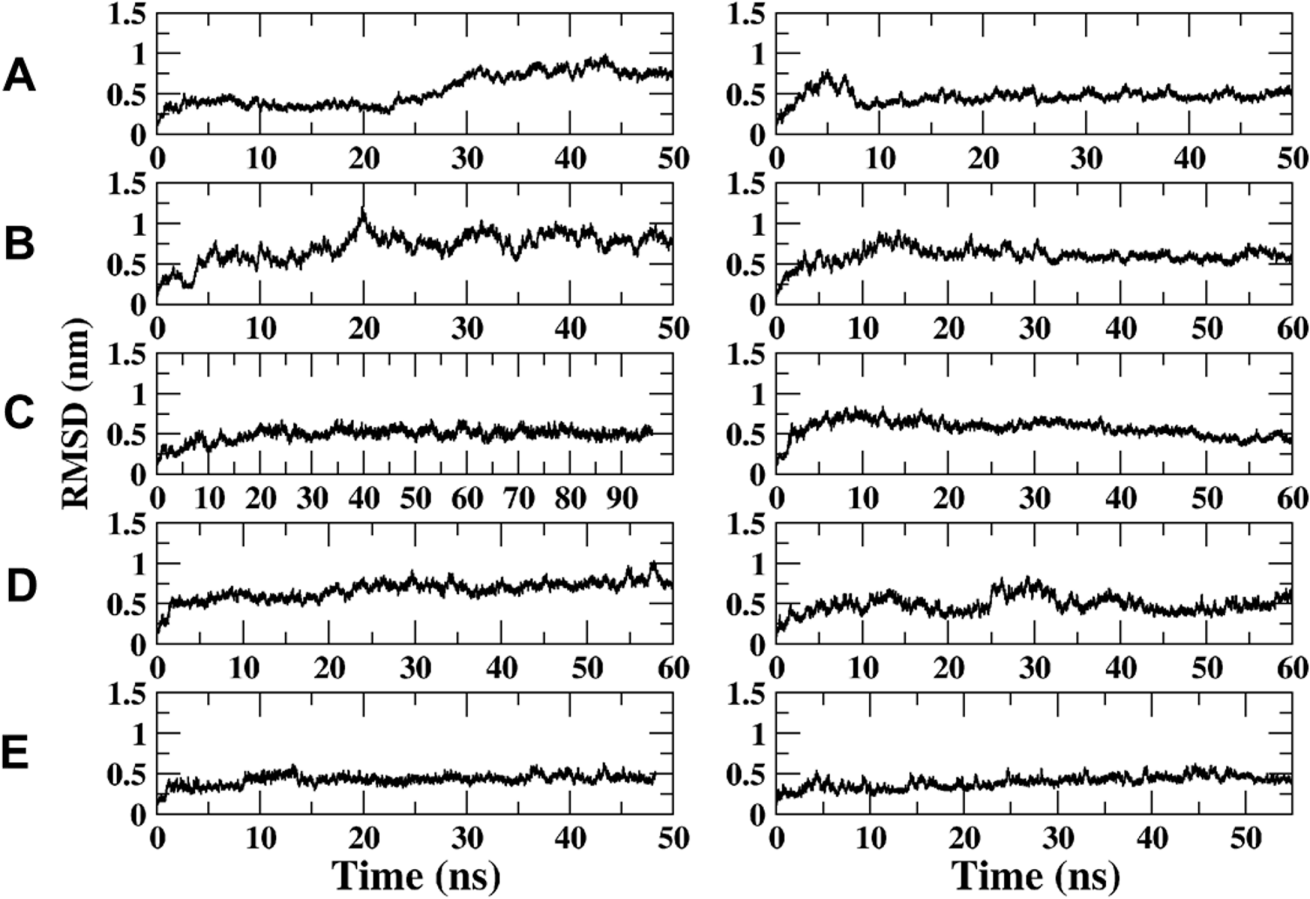
The RMSD of the Armadillo bound to FABP and the NLS-peptide in different orientation and location (major and minor site). (A) FABP4 bound in the major site parallel to the Armadillo (2 simulations—left and right); (B) FABP4 bound in the major site anti-parallel to the Armadillo (2 simulations); (C) FABP bound in the minor site parallel to the Armadillo (2 simulations); (D) FABP bound in the minor site anti-parallel to the Armadillo (2 simulations); (E) NLS peptide bound in both sites (major and minor) to the Armadillo; (F)–free Armadillo.

The fluctuations in the interaction energies, as calculated for the specific residues of the peptides reflect the motion of the charged moieties with respect to the Armadillo. To evaluate the extent of the motion of the backbone atoms we calculated the (geometric) average distance between each amino acid of the hepta-peptide and the residues of the Armadillo. Figs 6 and 7 represent those moieties where the average distance was less than 4 Å (marked in red) or closer, almost in touch, not to let a water molecule squeeze in (colored in blue). The analysis of the complex at the major site (Fig 6, frame A) implies that the moieties comprising the conserved NLS sequence and other residues maintain a close contact only with the Arms having the WxxxN repeats (Arms 2, 3 and 4). When attached to the minor site (Fig 6, frame B), the peptide experienced higher freedom and interacted with Arms 5 and 6 that do not carry the NLS recognizable domain in addition to Arms 7 and 8. Namely, at the major site, the interactions are only with the Arms that carry the WxxxN repeats, while at the minor site the NLS peptide is also stabilized by other Arms.

Based on these observations, the hepta-peptides-Armadillo complex seems to provide a "poor blueprint" for structuring the FABP4-Kapα complex. The structure of the hepta-peptides complex is constantly wiggling, the intensity of the interactions between the charged moieties of the NLS with the Armadillo fluctuate over time. Furthermore, residues that are not a part of the consensus sequence can make, in some time intervals, major contribution to the stability of the complex. Apparently, the freedom of motion while monitoring in aqueous solution at non-cryogenic temperature is much higher than what appears in the crystalline structure.

### The interaction of FABP4 with Armadillo

Protein-protein interaction differs from peptide-protein interaction by a lower density of hydrogen bonds. Upon binding, the short peptides lose their initial structure and expose their backbone atoms for hydrogen bonding with the protein [44]. Proteins, on the other hand, retain their internal structure and fewer hydrogen bonds can be made. Thus, the interaction of the FABP4 with the Armadillo is not a simple replication of the NLS peptide-Armadillo complex. To select which of the putative structures of the FABP4-Armadillo complex represents the transportable complex, we based our analysis on the conclusions resulting from the simulations of the Armadillo-SV40 NLS peptide: i. The contact between the two proteins can be as flexible as was noticed with the peptide. ii. The interactions may involve residues that are not *bona fide* members of the NLS sequence of the FABP4 (K21, K30 and R31) [8, 24] and the interacting Arms might include those not bearing the WxxxN sequence.

At present, as there is no direct evidence to which site on the Armadillo the FABP4 is bound, thus we had to consider both major and minor sites for their ability to bind the FABP4. The major site is generally considered to be the more attractive one [3, 4]. Yet it is known that in the case of the human phospholipid scramblase [11], the NLS sequence is made of only two basic residues and its binding site is the minor one. Considering the fact that one residue of the NLS sequence is separated from the others by a non-structured loop (K21, R30, K31 in FABP4 or K24, R33, K34 in FABP5 [10]), we could not exclude the possibility that actually only two residues participate in the binding and their preferred locus will be the minor site. What is more, there is no assurance that on forming a complex, the two proteins orient their N → C vectors in the anti-parallel mode as do the short NLS peptides. Consequently, both sites and two orientations of the vectors should be investigated to evaluate how the FABP4 protein interacts with the Kapα.

Four initial conformations were generated by the PatchDock server [23–25]. The setting of the proteins was such that at least two positive residues of the NLS of FABP4 were located close to the WxxxN bearing Arms (2, 3 and 4 for the major site) or Arms 7 and 8 (for the minor site), at a distance of ~ 5 Ǻ from the WxxxN moiety. We reasoned that such a separation distance can accommodate water molecules in-between the proteins, which ensures structural flexibility before tight contact is formed. Yet, at this proximity, the long-range non-bonded interactions between the proteins can still attract them one to the other and to form a tight contact.

The Docking process generated conformations where the N → C vectors of the two proteins are either in the parallel or the anti-parallel orientations. The highest scoring conformation of each orientation was selected as a starting structure for un-biased all atom molecular dynamics simulations, allowing the two proteins to adjust to the presence of each other. Each structure was simulated twice for 50 to 96 ns (Table 1).

**Table 1:** The simulations setup used for testing the interactions between the Armadillo with FABP loaded with linoleate. The length of the simulations is in units of nanoseconds. The directions of the N terminal→C terminal vectors of FABP4/Armadillo were either parallel or anti-parallel.

simulation	Major		Minor	
	parallel	anti-parallel	parallel	anti-parallel
1	50	50	96	60
2	60	50	60	60

Interestingly, some of the lower-scored structures generated by the PatchDock program were sampled during the early phases of the MD simulations.

#### Evaluation of the Stability and the Orientation of the Complexes

Fig 7 depicts the RMSD traces for the four simulated orientations of the FABP4 Armadillo complexes. These traces (A-D) are higher than the RMSD calculated for the SV40 NLS-Armadillo complex (Frame E) or the Armadillo without the peptides (Frame F). Thus, the FABP4 seems to lower the stability of the Armadillo.

**Fig 8:**
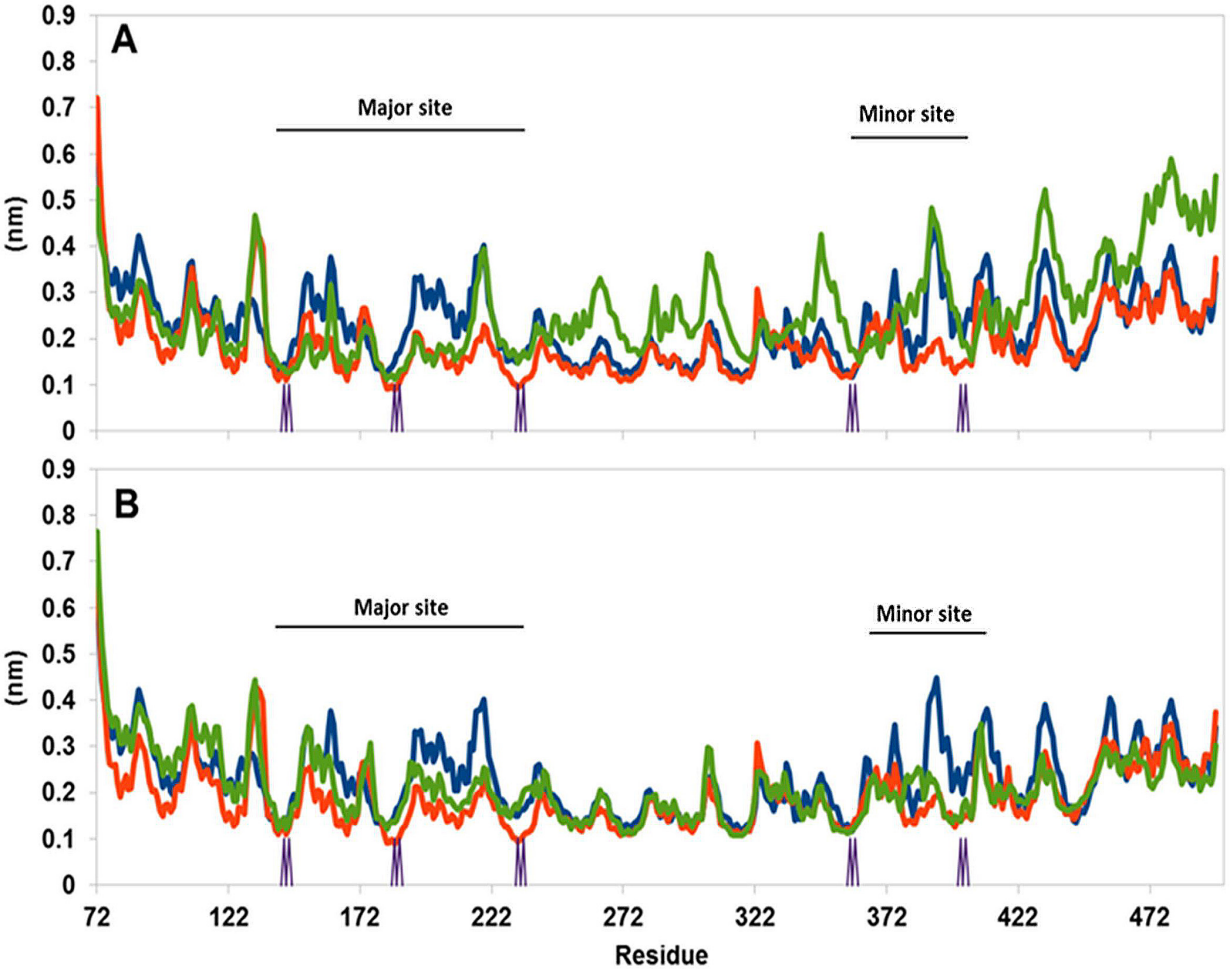
The RMSF of the FABP4-Armadillo complex as calculated for the parallel orientation. The simulation of the free Armadillo is presented by the blue line; the Armadillo bound to the NLS peptide is in red; the Armadillo bound to the FABP is in green. (A) The FABP4 is bound to the major site. (B) The FABP4 is bound to the minor site. The locations of the Armadillo's WxxxN repeats are marked on the x axis.

For a large protein made of repeating units (Arms) the variance of RMSD is less informative than examination of the RMSF of the specific residues (Fig 8). Frame A depicts the RMSF of the free Armadillo (blue trace), its complex with two SV40 NLS peptides (red trace) and the complex with parallel FABP4 at the major site (green trace). The most stable structure appears to be the Armadillo-NLS peptide complex. In this complex the RMSF values calculated at the major site are lower than those calculated for the free Armadillo. The complex with the FABP4 at the major site (green trace) shows similar behavior to that of the Armadillo-peptide complex, but this effect is limited only to the Arms forming the major site. From Arm 6 and on, the RMSF of the Armadillo with the FABP4 at the major site is significantly higher than the level set by the free Armadillo or its NLS complex. Frame B in Fig 8 (the same color code as in Frame A) depicts the RMSF calculated for the Armadillo with the parallel FABP4 docked to the minor site. When bound to the minor site, the RMSF trace (green) of the Armadillo exhibits stabilization both at the minor and the major sites, suggesting that the contact of the FABP4 with the Armadillo is not limited just to the minor binding site.

For the FABP4 complex with the Armadillo in the anti-parallel orientations, the RMSF values were much higher (data not shown).

**Fig 9:**
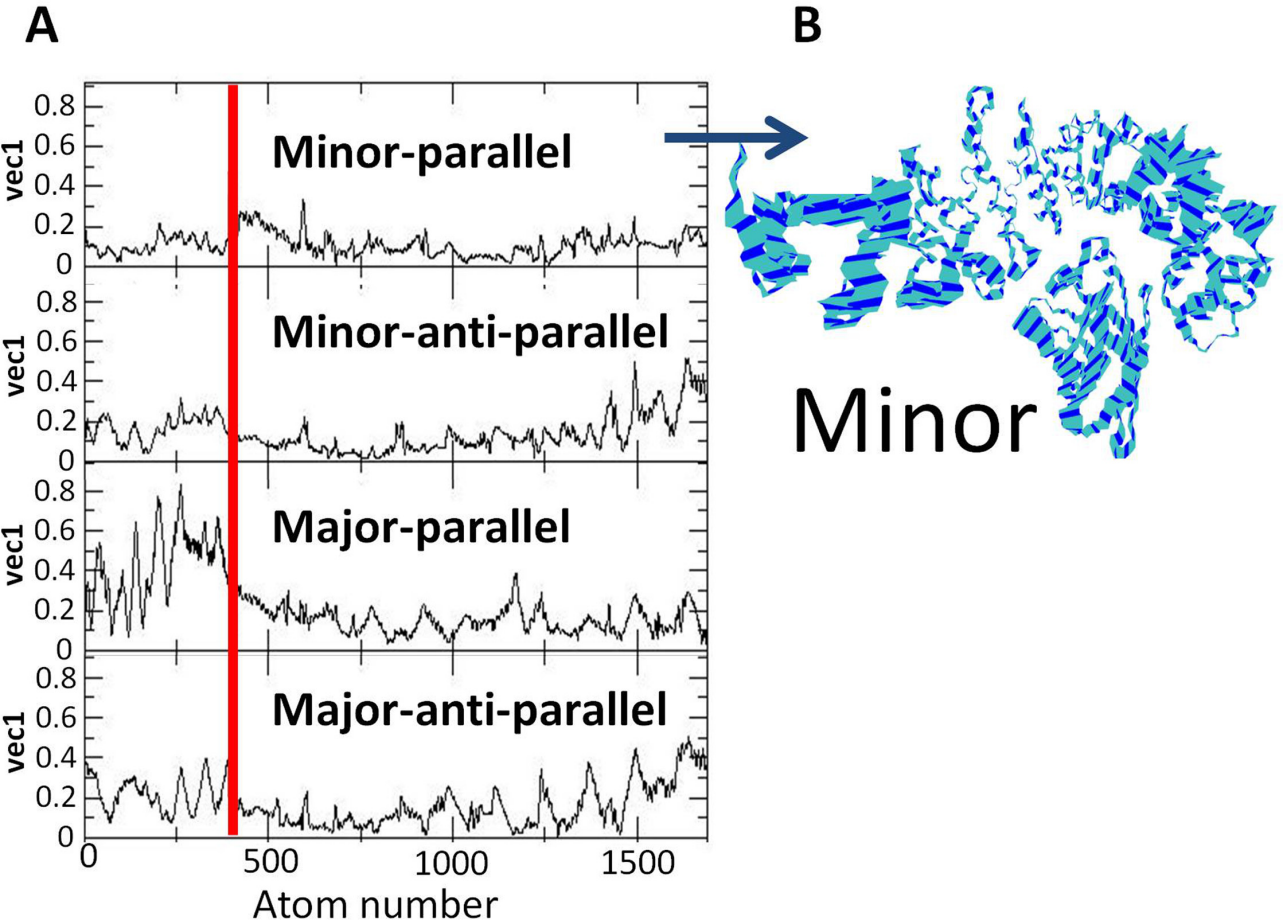
Principle Component Analysis (PCA) of the FABP4-Armadillo complexes. (A) The amplitudes of the largest vector (ordinate) as calculated for all heavy atom (abscissa) of the two proteins. The red vertical line delineates the FABP4 protein from the Armadillo. The orientation of each configuration is marked in the frames. (B) Presentation of the motion of the heavy atoms of the parallel-minor site alignment along the largest vector (wide stripes represent higher freedom of motion).

The trajectories of the four initial orientations were subjected to Principal Component analysis [42]; looking for the concerted motions that characterize the putative complexes. Fig 9, frame A depicts the amplitudes of the largest vector as calculated for the heavy atoms of the FABP4 and the Armadillo (drown sequentially on the abscissa). The upper panel, corresponding with the parallel-minor site orientation has the smallest amplitudes for both the FABP4 and the Armadillo. Frame B in Fig 9 presents the motion of the two proteins along the largest vector, as calculated for the parallel-minor configuration. This figure demonstrates how the interactions between the two proteins stabilize the contact region between them, while residues that are remote from the junction have higher freedom of motion. Comparison of the first vector of the four orientations reveals that the parallel alignment of the FABP4 next to the minor site generated the most stable complex and a visual projection of the first vector motion of the whole complex indicates that this configuration has the smallest structural fluctuation. Thus, the PCA calculations corroborate the conclusion that the most probable interaction between FABP4 and the Armadillo is the parallel docking at the minor site.

#### Interaction-Energy Analysis

**Fig 10:**
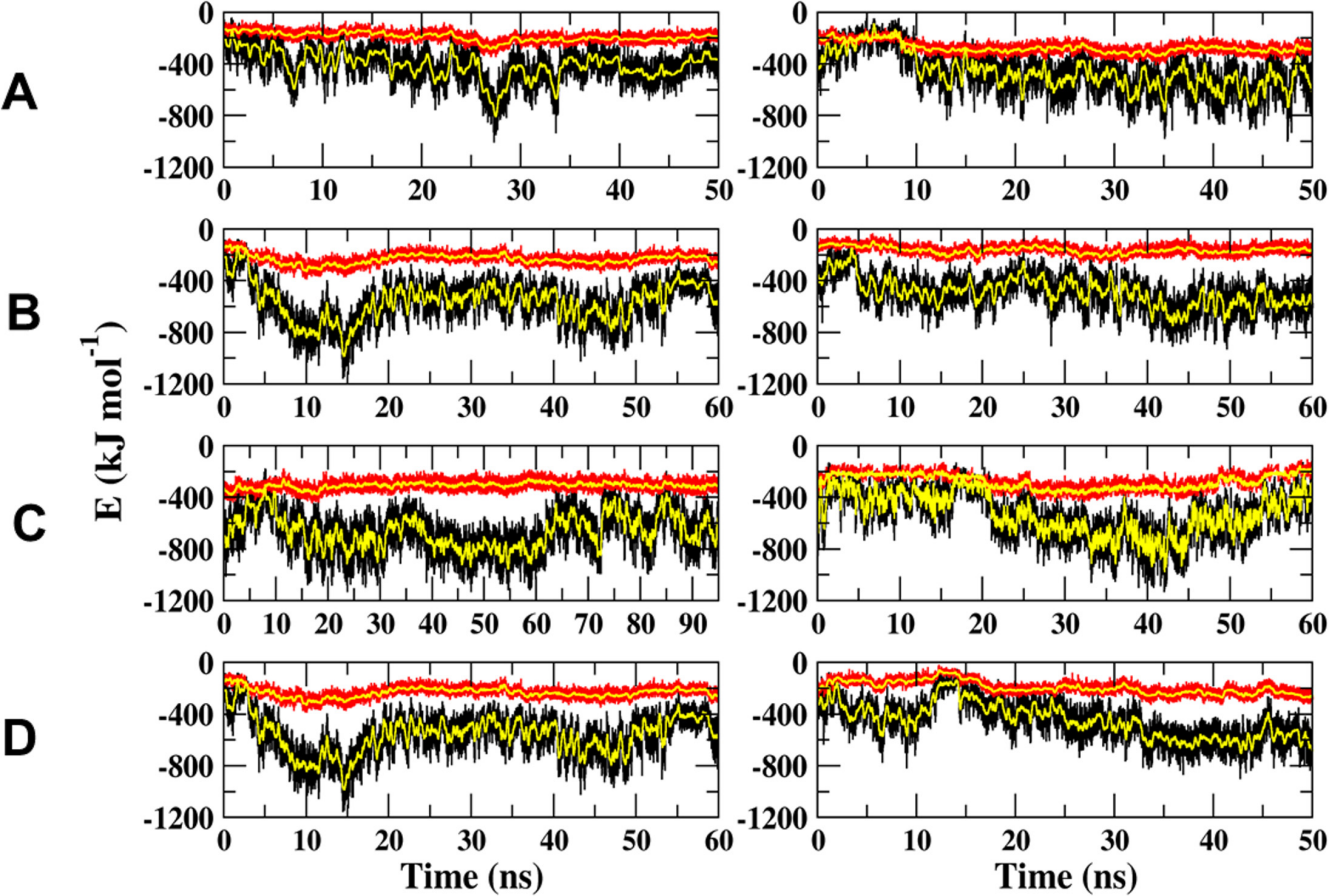
The interaction energies between FABP4 and Armadillo. The black and red traces represent the electrostatic and Lennard-Jones potentiale, respectively. The yellow traces are averaged values as calculated for a 100 ps wide time-window. The figure depicts, for each initial conformation, the outcome of two simulations (left and right Frames). (A) The FABP4 is bound at the major site in a parallel conformation. (B) The FABP4 is bound at the major site at the anti-parallel conformation. (C) The FABP4 is bound at the minor site at the parallel conformation. (D) The FABP4 is bound at the minor site at the anti-parallel conformation.

The interaction potentials (electrostatic and Lennard-Jones potentials) between the FABP4 and the Armadillo, as calculated along the whole length of the trajectories, are presented in Fig 10. The magnitudes of the Lennard-Jones interactions are similar for the four orientations and vary with time in a rather narrow range of -200 to -300 kJ/mol. The electrostatic interactions are fluctuating in time and vary in the range of -300 to -800 kJ/mol. The fluctuations in the electrostatic potential are attributed to relative motion of charged residues during the simulation time. These variations suggest that the two proteins are exercising a mutual probing process, where contacts are formed and replaced without reaching a permanent structure. The search mechanism represented by the oscillations of the electrostatic potential reflects the appearance of transient conformations that are in a rapid equilibrium. Such behavior can indicate that the system has not yet converged or, alternatively, that the nature of the contact between the two proteins is flexible and consists of rapidly alternating conformations. To discriminate between these two possibilities we carried out cluster analysis of the complexes and looked at the relative distribution of the clusters' sizes and the similarity between the shape of the dominating clusters and the temporal distribution of the clusters.

In order to sample the variety of the structures favored by the relaxed state of the complex, the analysis did not include the first 10 ns of the trajectories assuming that within this period the fluctuations include also some solvation events. On setting a cutoff value at 2 Å, the cluster analysis yielded for any of the simulations less than 50 clusters, where the largest one contained at least a few hundred structures; enough for statistical significance of the analysis. Comparison of the distribution of the clusters reveals that only FABP4 bound at the minor site in the parallel orientation reached convergence, and dominant structures can be clearly observed. The number of clusters calculated for the combined trajectories was smaller than the number calculated for each trajectory, indicating that both trajectories shared common structures. In the other three orientations the system failed to converge during the simulation times (data not shown).

**Fig 11:**
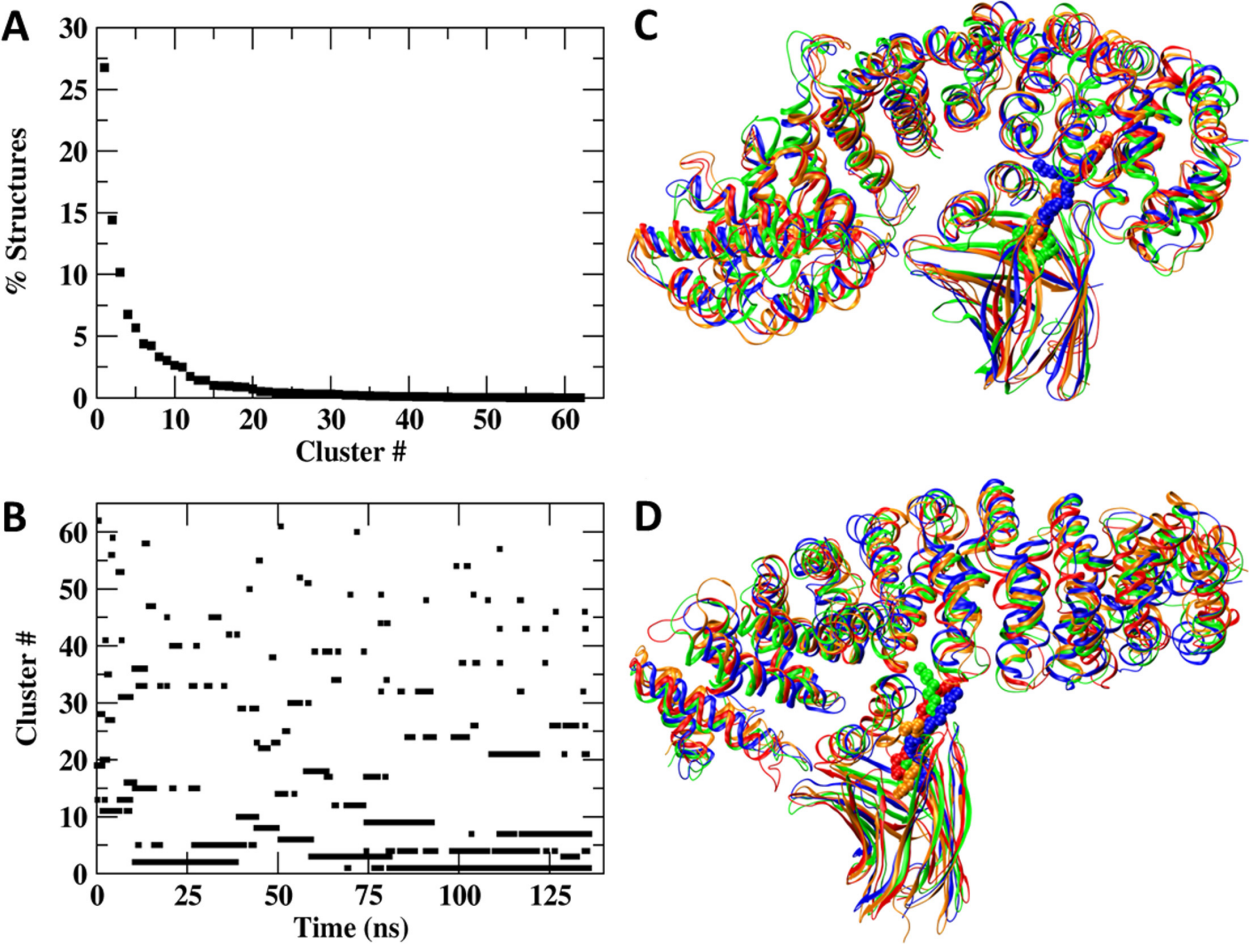
Cluster analysis of the combined trajectory of two minor parallel simulations of the FABP4-Armadillo complex. (A) depicts the size distribution of the clusters. (B) depicts the time distribution of the clusters. The data relates the cluster number with the time points along the trajectory. (C) presents superposition of the four largest clusters (each cluster has a different color) of the minor parallel simulation. Please note that even after the dominant structure had converged there are brief appearances of low probability states. (D) depicts, for comparison, the four largest clusters (each cluster has a different color) of the major parallel simulation.

The analysis of the combined trajectory presented in Fig 11 reveals that the cluster size (Frame A) decreases in a smooth curve, where the ratio between the size of consecutive clusters indicates a free energy difference ($\Delta G={\text{-}k}_{B}\text{T}\times \ln\frac{{\text{cluster}}_{\text{i}}}{{\text{cluster}}_{\text{j}}}$) between consecutive clusters (cluster_i_ /cluster_(i+1)_) to be in the order of 1*k*_B_T (or less than that). The temporal distribution of the clusters (Frame B) indicates that during the whole length of the trajectory, the stable structures can temporarily gain a transient less stable conformation. This indicates that the complex between the two proteins is a dynamic structure that constantly samples possible conformations that are in fast thermodynamic equilibrium.

The structural similarity between the clusters was evaluated by superimposition of the four largest clusters, representing ~60% of all conformations sampled by the simulations. Fig 11, frame C represents the four largest clusters of the FABP4 bound in the parallel orientation at the minor site. The rigid sections of the protein retain their shape and orientation, while the variance is mostly at the loops connecting the helices and the β strands. The interaction between the FABP4 and the Armadillo is not limited to residues located at the lid and the Arms of the minor site. As shown in frame C the β strands of the FABP4 make contact with Arms 1–5, where the major site is located. These interactions may explain how the binding at the minor site enhances the stability of the major site (Fig 8 above).

Fig 11, frame D depicts the four largest clusters of the FABP4 attached in the parallel orientation at the major site. In this case, there are no interactions with the minor site, accounting for the higher RMSF of the minor site in this complex (Fig 8 above).

**Fig 12:**
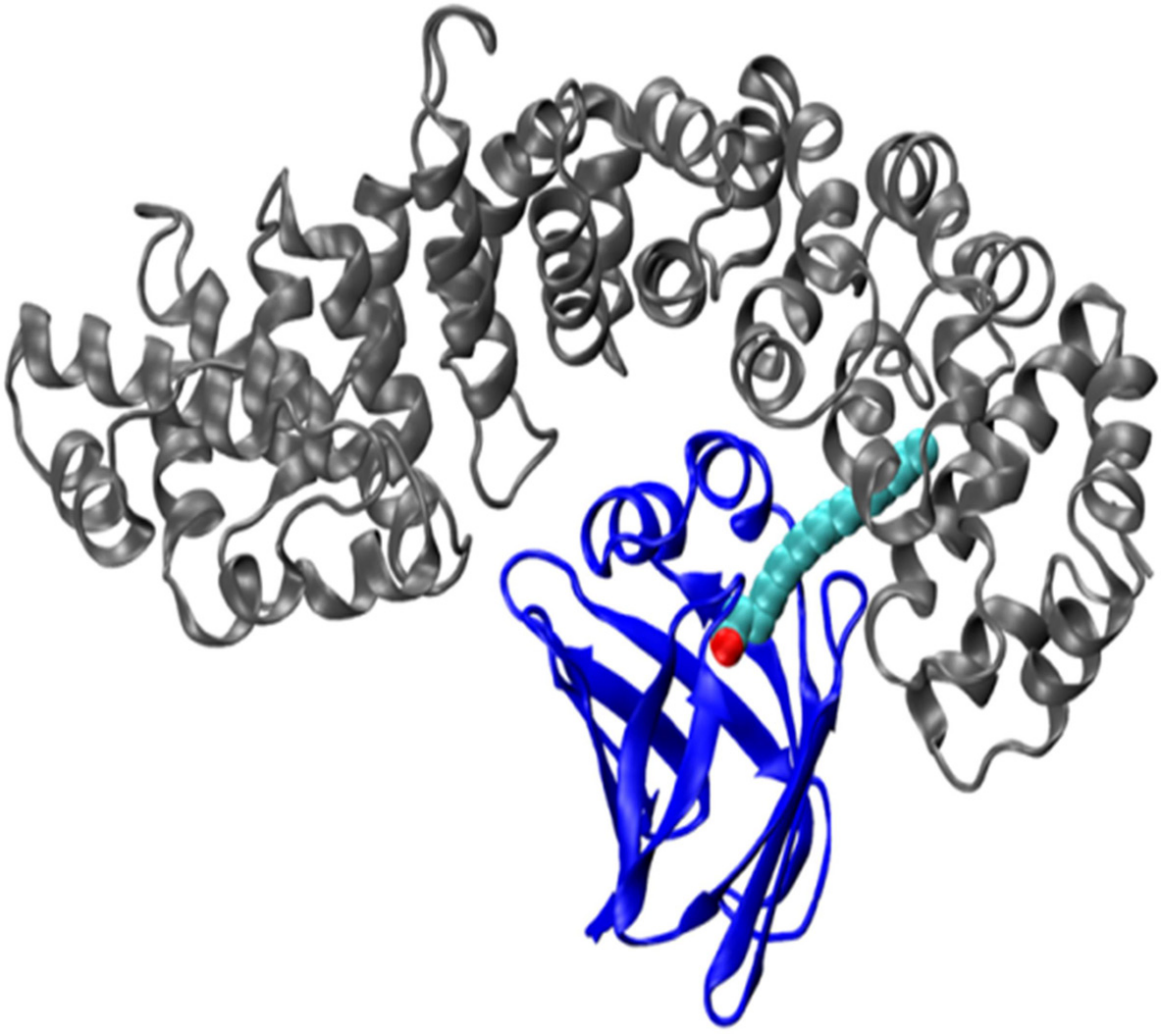
Representation of the largest cluster of the complex of FABP4-Armadillo at the minor site. The Armadillo (gray) and FABP4 (blue) are shown in new-cartoon style, while the linoleate molecule is represented in VdW radii. The linoleate molecule shifts from the initial position to a new location where it is held both by the FABP4 and the Armadillo.

Upon simulations of the FABP4 complex at the minor-parallel orientation, we noticed a peculiar behavior that was never observed before: the linoleate molecule was gradually migrating from its original binding site inside the FABP4 molecule to a new location at the interface between the two proteins. In this conformation, the hydrophobic section of the fatty acid is embedded in the crevice between Arms 8 and 7 of the Armadillo (Fig 12). This sharing of ligand between FABP4 and Armadillo was not noticed in any other trajectory of the FABP4-Armadillo complex. It should be mentioned that simulations of the FABP4 by itself, loaded with the linoleate, did not reveal any tendency of the fatty acid to migrate out of the hydrophobic pocket of the protein (Ortal Amber-Vitos, unpublished results).

**Fig 13:**
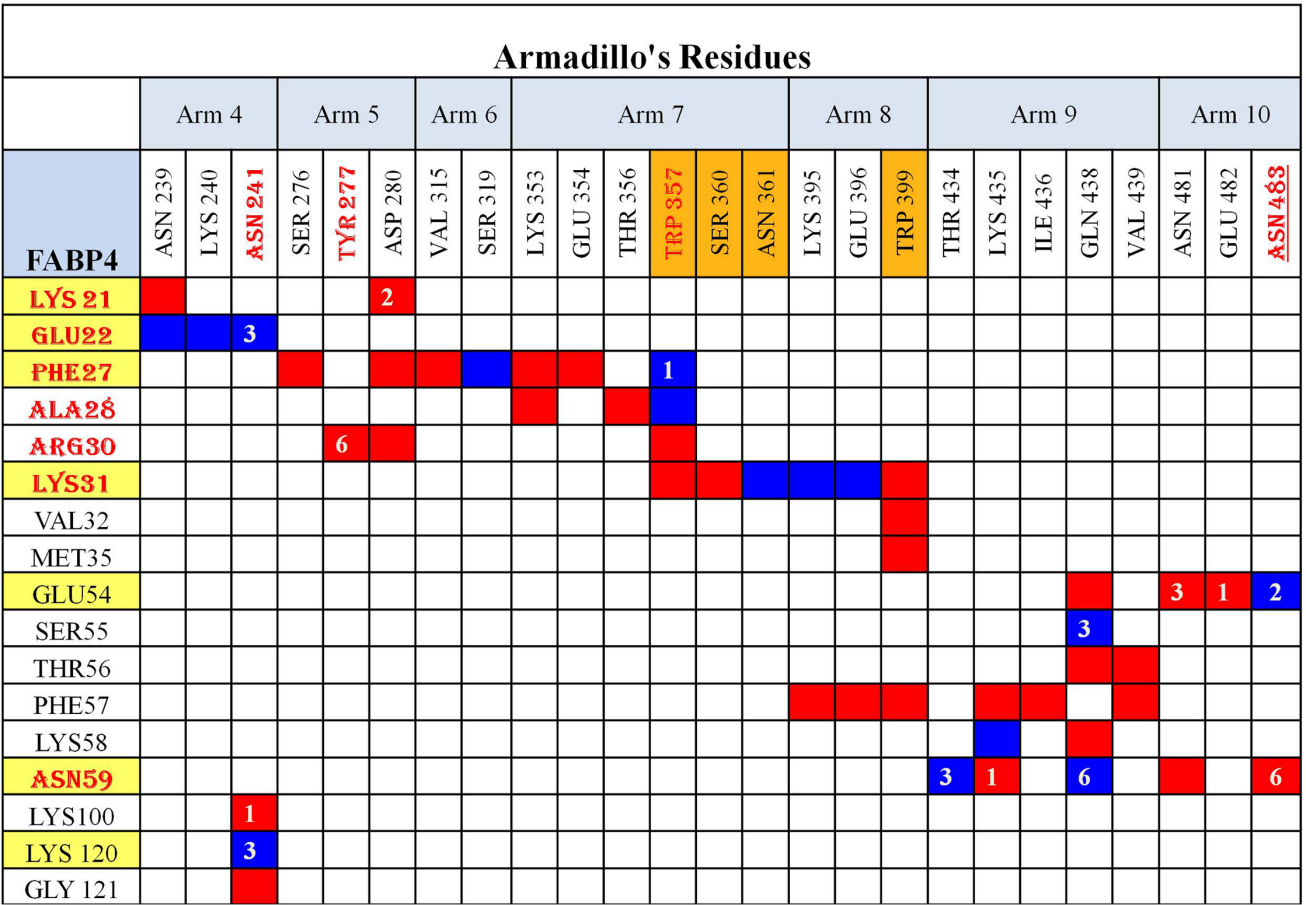
The most proximal pairs in the FABP4-Armadillo complex at the minor site in parallel orientation. The table lists all residues on the FABP4 and on the Armadillo for which the (geometric) average of the distance between them was less than 4 Å (marked in red) or less than 2.8 Å (blue). Residues on FABP4 marked with yellow are those that make a significant contribution to the stabilization energy of the complex. Residues of the Armadillo colored in orange are members of the NLS recognition sites (WxxxN). Residues marked in red are identified as Hot Spots [41]. The number of hydrogen bonds formed between two residues [43] is given in the table.

The consistency of the contact between the two proteins in the complex was evaluated by the calculation of the (geometric) time average distance between pairs of residues located on the two proteins. The data in Fig 13 shows (in red) pairs that were less than 4 Å apart and (in blue) residues that are too close to accommodate a water molecule between them.

For the FABP4 located at the minor site of the Armadillo in the parallel orientation, there are 45 time-stable contact points where the separation between the residues is less than 4Ǻ. These interactions include both “consensus” residues (located on the NLS) as well as interactions far from the NLS interaction domain. Some are made by charged residues (E22, E54, K58, K100 and K120) and others are polar (S55, T56 and N59) or even hydrophobic in nature (F27, A28, V32, F57 and G121). These contacts involve not only the WxxxN Arms of the minor site (Arms 7 and 8), but also Arms 4, 5, 6, 9 and 10. Thus, at this location, the FABP4 forms an extended network of interactions with the whole length of the Armadillo. The spreading of the contact points on both binding sites of the Armadillo issues the enhanced bending of the Armadillo, which brings the FABP4 closer to the major site. In this mode of interaction, the FABP4 at the minor site blocks the major site from reacting with the IBB domain of the Kapα, enhancing the propensity of the IBB domain to react with the Kapβ [11], thus forming the structure that can permeate the Nucleopore.

The conclusions derived from the time averaged distance between residues were supported by the Hot Spots analysis [41]. The analysis was carried out for the dominating clusters of the four different simulations. In the case of the minor-parallel orientation, the three NLS (K21, R30, K31) residues and E22, F27, A28, N59 of the FABP4, together with N241, Y277, W357 and N483 of the Armadillo were identified as Hot Spots (marked in Red-Bold letters in Fig 13). A systematic analysis of hydrogen bonds between the two proteins identified 36 hydrogen bonds, but only 6 of them involved the NLS moieties of FABP4 (K21 and R30). The other hydrogen bonds involve non-NLS residues, some of which are not even part of the lid (the numbers of hydrogen bonds with the Armadillo are given in Fig 13).

When the time average distance between pairs of residues analysis was carried out for the other orientations, the number of tight contacts was significantly (~50%) smaller and each of the simulations yielded a different set of “tight” contacts (data not shown). The Hot Spots analysis of the largest clusters of these orientations failed to identify some (or all) of the NLS residues (data not shown). Furthermore, the number of hydrogen bonds [43] that were detected for the other orientations were much smaller compared to those found for the minor parallel configuration (20 hydrogen bonds for the minor anti-parallel; 19 for major anti-parallel and 10 for major-parallel).

**Fig 14:**
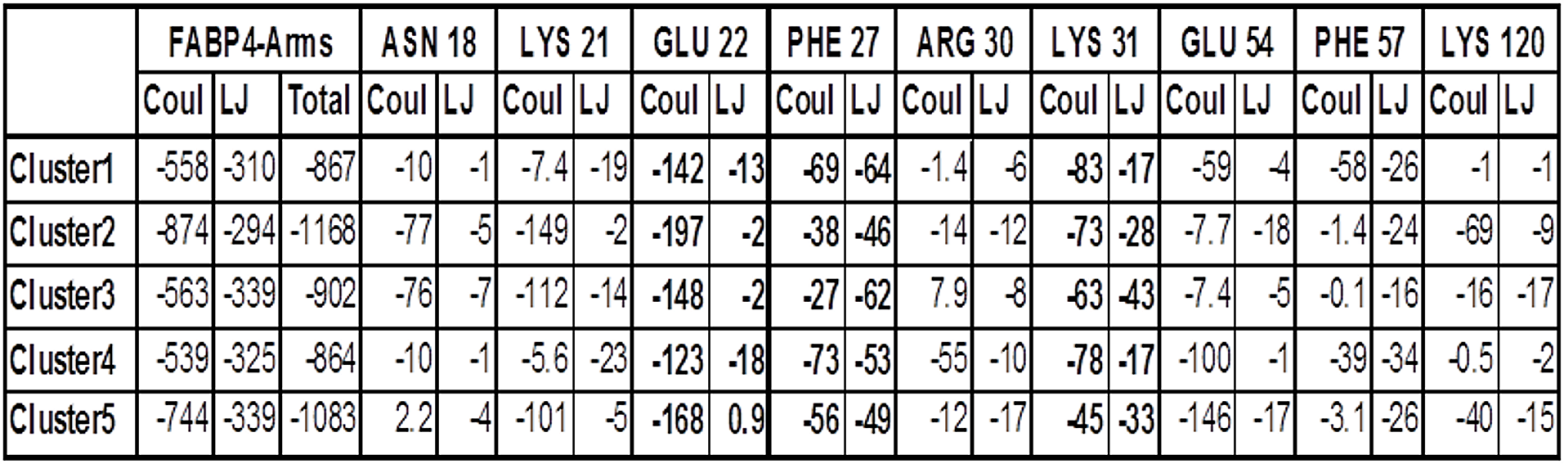
The interaction energies between the residues on FABP4 with the Armadillo. The data were calculated for each of the five largest clusters representing the complex FABP4 (loaded with linoleate) located at the minor site in the parallel orientation.

The contributions of the residues of the FABP4 to the interaction-potential of the two proteins were calculated for the representative structures of the five largest clusters of the minor parallel complex. Fig 14 details, for each cluster, the values of the Lennard-Jones and electrostatic potentials for each of the interacting residues of the FABP4. Some of the residues (bold numbers) make comparable contributions in all clusters (e.g. E22, F27 and K31), while the interaction energies of others vary by factors of 10 between the clusters (K21, R30 and F57). Apparently, as with the NLS peptides-Armadillo interactions, the complex is flexible in nature, performing the same type of “step dancing” in which the contact is permanently maintained but the relative contributions of the many contacting residues vary with time.

As appears from the present study, the interaction between the FABP4 and Armadillo utilizes many residues located on the lid structure. These interactions are noticed only for the specific orientation (minor-parallel), where the lid seems to lose its tight structure. The ability of the lid to relax, and the correlation between the flexibility of the structure with the nature of the ligand bound to the FABP4 was investigated by the application of Constraint-Network Analysis (CNA) [36], a server that calculates the thermostability of the various structural elements of a protein.

**Fig 15:**
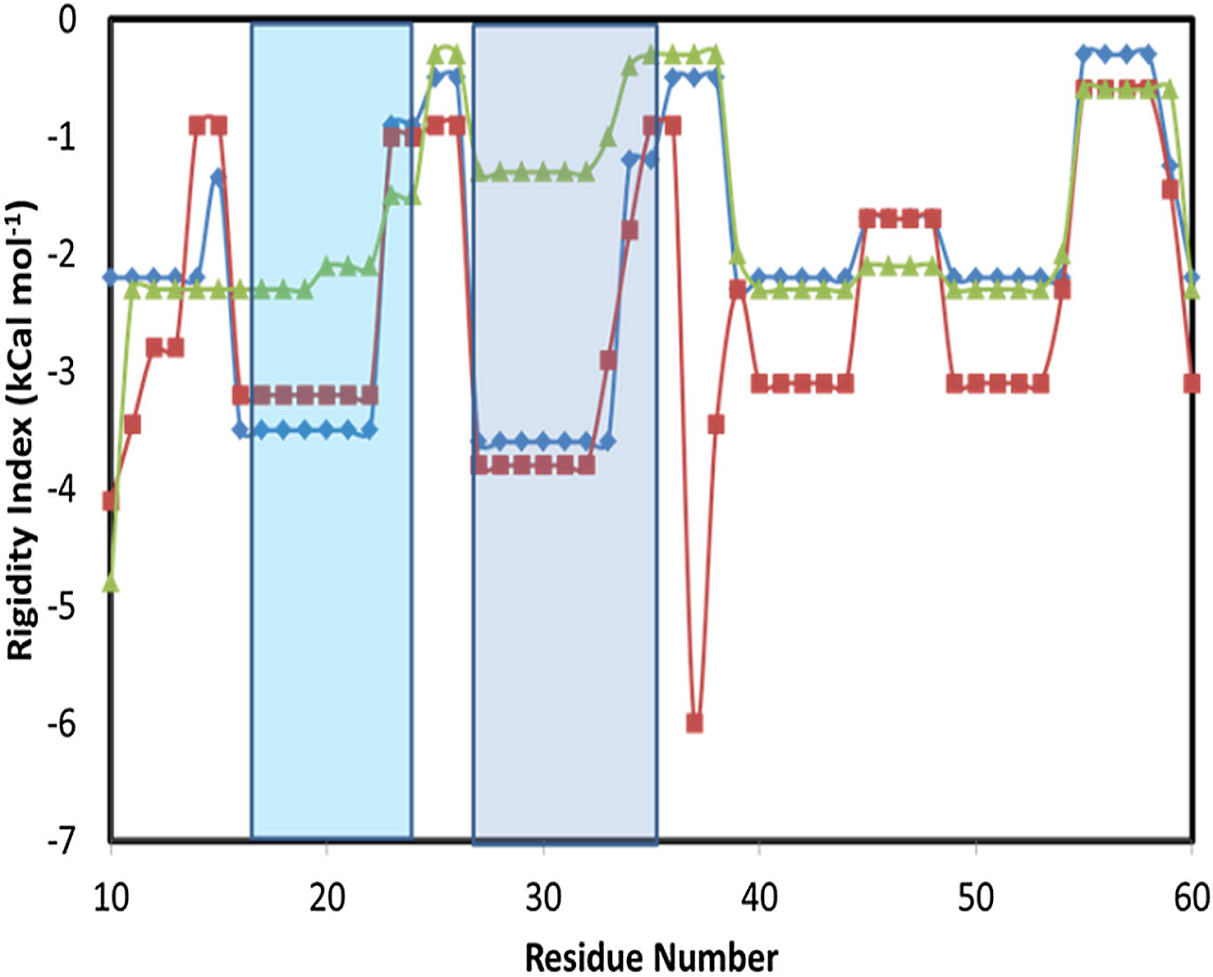
Rigidity index of Cα atoms for residues 10–60 of the FABP4 in different crystal structures. The blue line represents the Apo form of FABP4 (1ALB.pdb), the red line represents a complex with palmitate (1LIE.pdb) (both are non-transportable compounds) and the green line represents a complex with linoleate (2Q9S.pdb). The two helices (I and II) forming the lid are highlighted in light and dark blue, respectively.

Fig 15 depicts the thermostability of FABP4 in its Apo state and in a complex with non-transportable ligand *vs*. a complex with linoleate. For simplicity, the presentation is limited only to residues 10–60, where the two helices of the lid are located. The residue number is indicated on the x-axis and the y-axis denotes the rigidity index of the Cα atoms in kcal/mol [37]. What is well perceived from this figure is that the rigidity of the two helices, calculated for the transportable complex (the FABP4 loaded by linoleate) is significantly smaller than that of the non-transportable protein (Apo and palmitate-loaded). In the complex with linoleate, the helices of the lid are rather flexible and tend to depart from the rigid helical structure. Repeating the same analysis for two more transportable substrates (ANS (anilinonaphthalene sulfonate) (2ANS.pdb) and TDZ—(Troglitazone) (2QM9.pdb)) reveals also low rigidity of the helices, while for the non-transportable compounds (Archidonate—1ADL.pdb, Oleate—1LID.pdb, stearate—1LIF.pdb and hexadecanoate—1LIC.pdb) all exhibited high rigidity of the lid domain (data not shown). Apparently, the flexibility of the lid domain may play an important role in the recognition mechanism by the Kapα.

## Discussion

The classical monopartite NLS domain is made of three basic moieties conforming to the consensus sequence KK/RXK/R. The studies of their mode of interaction with the Karyopherin system is based on crystallization of the Kapα^ΔIBB^ (the Armadillo without the IBB domain) with short segments of the imported proteins, up to a length of ~20 residues [45]. These crystals reveal a complete structure-less peptide that interacts tightly with the Armadillo using both side-chain and backbone atoms to form a stable crystalline complex. In the present study, we investigated the possible nature of a complex between a native protein with Kapα—a complex where the shape of the NLS domain is affected by the internal interactions of the ligand-loaded FABP.

The simulations of a *bona fide* classical monopartite short peptide revealed that the complex is much more flexible than appears from the crystal structure. The dynamics of the short, structure-less peptide revealed that while the backbone atoms were quite persistent in their location (especially when bound at the major site), the polar and charged moieties were highly mobile, moving from locations where their electrostatic potential was ~ -200 kJ/mol to sites where their hydration practically nullified their interaction with the Armadillo. These fluctuations are much larger than the B factors calculated from the crystal. Another feature, revealed by the simulations, was the stabilizing contribution of residues that are not part of the classical NLS consensus, namely proline and valine. Based on these observations it seems that the dynamics of the peptide-Armadillo complex could not serve as a model to predict the precise shape of the cryptic NLS of FABP4 complex with the Armadillo.

From the simulations of FABP4 loaded with linoleate, tested in the present study, only one mode of contact (parallel orientation in contact with the minor site) out of the four possible conformations appeared to present a persistent complex between the two proteins. The complex exhibited a large number of contact points, tight enough to retain an average distance of less than 4 Å during a simulation time of 96ns, this in comparison with the other conformations in which the number of contacts was 50% or less. In this conformation, all residues of the NLS (K21, R30, K31) were identified as Hot Spots, two of them (K21 and R30) together with some non-NLS moieties (E22, F27, K100, K120 and G121) formed tight lasting contact with the Armadillo including Arms 4, 5 and 6 that are components of the major site domain. This special conformation, in which the FABP4 interacts with both the minor and the major sites, prevents the IBB domain from folding over the major site and rendering it free to react with Kapβ.

At first glance, there seems to be some internal contradiction in the present study: the structure of the NLS peptide-Armadillo complex utilizes many more interaction sites than the NLS domain of the FABP. This apparent discrepancy is a direct reflection of the basic difference between the mode of a short peptide binding *vs*. the binding of a well folded protein [44]. The peptide has the freedom to sample a large variety of conformations that are almost equipotential [35]. Thus, it can easily assume a shape that will mostly fit into a rigid binding site of a protein. As a results, the protein-peptide complexes are richer in hydrogen-bond interactions (most of them with the backbone atoms) than the protein-protein complexes [44]. This ability, to fit tightly into a binding site, is denied from a protein due to its inherent structure. In the case of FABP4, where the NLS site consists of residues located on two adjacent helices, the nature of the complex will diverge from that of the peptides-Armadillo complex. The positively charged residues; K21, R30 and K31, do serve as primers of the docking that are attracted to the crevice of the binding site. But the nature of the mature complex has more features of a protein-protein complex than of a protein-peptide one.

Recently, Pang and Zhou [46] investigated the interaction of short peptides with the minor site, demonstrating the significance of the interaction of two adjacent positive moieties to the binding. Their modeling indicated the role of non-polar interaction of other moieties of the NLS sequence with nearby Arms, even with those of the major site. Their study, with a model peptide, clearly confirm our suggestion that in the case of protein-protein interaction, where the NLS sequence is located on a well-structured domain, the stabilization is gained by interactions that are beyond the formalistic NLS residues.

Finally, the minor-parallel conformation generated an extended hydrophobic domain which expanded out of the boundary of the FABP4, letting the load carried by the FABP4 (linoleate) to partially migrate out of the FABP4 protein into a mutual complex, in which the Armadillo contributes part of a joint binding pocket. It might be suggested that such a complex is instrumental in the liberation of the cargo into the nucleus space, once the loaded FABP4 reaches the inner space of the nucleus.
